# Primary Care Provider Receptivity to Multi-Cancer Early Detection Test Use in Cancer Screening

**DOI:** 10.3390/jpm13121673

**Published:** 2023-11-29

**Authors:** Christopher V. Chambers, William T. Leach, Kaitlyn Davis, Ronald E. Myers

**Affiliations:** 1 Department of Family and Community Medicine, Thomas Jefferson University, Philadelphia, PA 19107, USA; william.leach@jefferson.edu (W.T.L.); kaitlyn.davis@jefferson.edu (K.D.); 2 Division of Population Science, Department of Medical Oncology, Thomas Jefferson University, Philadelphia, PA 19107, USA; ronald.myers@jefferson.edu

**Keywords:** multi-cancer early detection tests, primary care, cancer screening

## Abstract

Multi-cancer early detection tests (MCEDs) are blood-based tests that detect biomarkers released or induced by cancer cells. If MCED tests are shown to be safe and effective in cancer screening, they are likely to be ordered and managed in primary care. To understand primary care providers’ support for and concerns about the implementation and management of MCED testing, the research team developed a cross-sectional survey that was sent to 939 primary care providers (physicians, residents/fellows, and advanced practice providers) in a large academic health system in the greater Philadelphia area. The survey included standard items used to assess provider background characteristics and to measure provider awareness of challenges related to MCED test use (7 items), perceived competence in MCED testing (5 items), and receptivity to MCED test use in the future (4 items). A total of 351 (37.4%) primary care providers completed the survey. Among respondents, the awareness of challenges in MCED testing (mean = 3.95, sd = 0.64), perceived competence (3.67, sd = 0.85), and receptivity to MCED use in practice (mean = 3.62, 0.75) were moderately high. Multiple regression was performed to identify factors associated with receptivity to MCED testing. We found that provider number of years in practice (DATA), awareness of challenges related to MCED testing (DATA), and perceived competence in MCED test use (DATA) were positively and significantly associated with receptivity to MCED test use in practice. An exploratory factor analysis extracted two components: receptivity to MCEDs and awareness of challenges. Surprisingly, these factors had a positive correlation (r = 0.124, *p* = 0.024). Providers’ perceived competence in using MCED tests and providers’ experience level were significantly associated with receptivity to MCED testing. While there was strong agreement with potential challenges to implementing MCEDs, PCPs were generally receptive to using MCEDs in cancer screening. Keeping PCPs updated on the evolving knowledge of MCEDs is likely critical to building receptivity to MCED testing.

## 1. Introduction

Cancer remains one of the leading causes of death for adults in the United States (U.S.) [1]. The American Cancer Society projects that in 2023, there will be almost 2 million new cancer diagnoses and over 600,000 deaths [2]. While most cancers are more amenable to treatment when detected in early stages, standard of care (SOC) cancer screening is currently recommended for only four cancers (breast, cervical, colorectal, and lung) [3,4,5,6]. Approximately 70% of cancer deaths are caused by a cancer for which there is no screening test that has been shown to reduce cancer mortality and is recommended for use in routine care [7]. In addition, some of the screening tests have barriers that prevent broad uptake such as the preparation associated with colonoscopies or the exposure to radiation with low-dose computed tomography (CT) scans or mammograms [7,8].

To address these gaps in cancer screening, several companies across the U.S. are developing multi-cancer early detection (MCED) tests [9,10]. MCED tests are blood-based tests that detect biological signals, or biomarkers, that are either released by cancer cells or induced by their presence. In the future, safe and effective MCED tests may be recommended for use in concert with current SOC cancer screening [11]. MCED blood tests currently in development can detect from 3 to over 50 different cancers with a single blood sample collected using a standard venipuncture procedure [9,11]. These tests also hold promise that the cancers detected will be identified in earlier stages when they are more amenable to treatment options including curative surgery. While few MCED clinical trials have been completed to date (DETECT-A [9], Circulating Cell-Free Genome Atlas (CCGA) [11], SYMPLIFY [12]), more are expected to not only demonstrate safety and efficacy, but to also be examined for clinical implementation.

Standard of care cancer screening is typically managed by primary care providers (PCPs). In this context, PCPs are typically required to place a screening referral or order a screening exam for patients who are eligible for cancer screening [13]. As companies prepare to bring their MCED tests to market and make them widely available, it is important to understand the level of support from PCPs and what concerns they may have related to such testing (e.g., managing MCED results). In the study reported here, we assessed the receptivity, concerns, and perceived competence of PCPs utilizing MCED testing in clinical practice.

## 2. Materials and Methods

### 2.1. Study Design

We conducted a cross-sectional survey of PCPs within an academic health system in a large metropolitan area to assess their perceptions about MCED use for primary care patients and their support for participation in an MCED clinical trial. The study was conducted from June 2022 to February 2023. This study was reviewed and approved by the Institutional Review Board of Thomas Jefferson University.

### 2.2. Participants

To identify participants for the survey, the study team worked with the health system’s Human Resources (HR) department. At the time the study was conducted, the health system included 98 primary care practices of various sizes (range of 3–38 providers) servicing patient populations that are diverse with respect to race, ethnicity, and insurance coverage. In June 2022, HR generated a list of all PCPs (staff physicians and mid-level providers) that were active in a primary care practice within the health system. The team reviewed the list with the guidance of PCPs on the team to ensure that only those actively seeing patients were invited to participate in the survey.

The initial survey invitation was sent to all identified PCPs in July 2022. A few days before the survey invitation was sent, the medical director at each of the system’s six campuses emailed their providers to alert them that a survey invitation would be coming. Each provider received up to 4 reminders if they did not complete the survey by the time the survey period closed in December 2022.

We also worked with HR to produce a list of all primary care trainees (i.e., residents and fellows) in the health system to be targeted for survey administration. A similar workflow was followed to administer the same survey to them. Residency and fellowship program coordinators at each campus helped the study team ensure that the list from HR was complete. The initial survey invitation was sent to trainees in December 2022. A few days before the invitation was sent, residency program coordinators emailed the trainees in their program to alert them that an invitation was coming. Each trainee received up to 4 reminders if they did not complete the survey by the time the survey period closed in February 2023.

### 2.3. Procedures

This study utilized an exploratory survey that initially described a planned MCED clinical trial designed to evaluate the safety and efficacy of an MCED test for cancer screening. The survey explained that trial participants would be 50 to 84 years of age, participants would complete an MCED blood test, and those who had an abnormal test result would be advised to have a PET-CT diagnostic examination. In this section of the survey, the providers were asked to respond to items designed to elicit their views related to supporting patient participation in the planned trial. The findings from the analyses of the data collected in this section are not presented in this report.

In terms of MCED testing outside the context of the planned clinical trial, the survey included a subsequent section that asked PCPs to assume that the clinical trial had been completed and the Food and Drug Administration (FDA) had approved the MCED test for clinical use. Given this scenario, providers were asked to respond to 11 Likert-type response items (0 = Strongly Disagree to 5 = Strongly Agree) that were designed to determine the attitudes and beliefs related to use of the FDA-approved MCED test in practice. These survey statements were adapted from a questionnaire administered to primary care physicians in a prior study designed to determine the PCP views on genetic testing [14].

This section of the survey also included five Likert-type response items (0 = Strongly Disagree to 5 = Strongly Agree) adapted from the Perceived Competence Scale [15,16]. Specifically, respondents were asked to indicate their level of confidence in interacting with patients about MCED testing which included educating patients about MCED testing, engaging patients in shared decision making about MCED testing, explaining false positive MCED test results, explaining false negative MCED test results, and guiding patients through diagnostic evaluations to follow up on an abnormal MCED test result.

The survey also included a set of items that asked respondents to provide details on their background characteristics. That is, this section of the survey allowed respondents to report their age, gender, race, ethnicity, clinical degree, years in practice, board certification status, and medical specialty.

### 2.4. Data Analysis

Demographics, board certification, and provider experience were summarized using frequencies with percentages and mean (SD)for categorical variables.

The primary outcome was based on the extracted factor representing provider receptivity. Briefly, we utilized exploratory factor analysis (EFA) to analyze the experimental section of the questionnaire and understand its factor structure. We hypothesized that the factor extraction for the items in the exploratory questionnaire would result in a single factor representing provider receptivity to MCED use. However, convergent evidence was found for a two-factor structure using the Kaiser criterion, scree plot, and parallel analysis [17,18,19,20]. The method of factor extraction was generalized least squares, and an oblique rotation (Direct Oblimin) was applied given the correlations present between items. The first factor was comprised of four questions and termed “receptivity” and represented providers’ willingness to order an MCED test and beliefs regarding the benefits of MCED testing. The second factor was comprised of six questions and termed “awareness of challenges” as it represented potential challenges to the use of MCED tests, such as concerns about false positives and negatives, and the time and cost for interpretation and following up on MCED results. The internal reliability of each factor was calculated using Cronbach’s alpha. Given this two-factor structure, we analyzed the data in terms of subscales (i.e., “receptivity” and “challenges”) instead of an aggregate score from the 11-item exploratory questionnaire with the receptivity subscale as our primary outcome. 

With two factors extracted, subscale scores were calculated for both the receptivity and challenges factors by averaging the included items. A score was also calculated for the perceived competence in test use scale. Score means and reliability coefficients were established. 

Multiple regression was used to test if providers’ receptivity to using MCEDs could be predicted by providers’ perceived competence in using MCEDs and providers’ experience. One aspect of great interest was that our sample was comprised of staff physicians and trainees (i.e., residents and fellows). A categorical variable representing PCP experience was created using the median years in practice. Three levels were created: trainees (residents and fellows), staff physicians (under 18 years in practice), and experienced staff physicians (18+ years in practice). APPs were excluded from this analysis as they receive different training and were a relatively low portion of our sample (6.8%). All analyses were performed using SPSS version 29.0.

## 3. Results

### 3.1. Demographics

To understand PCPs’ perceptions of MCED tests and their use, 939 providers were invited to complete the survey (Table 1). A total of 351 PCPs responded (37.4%), including 124 staff physicians (36%), 201 residents and fellows (58%), and 24 advanced practice providers (7%). The average age of respondents was 39.12 (σ = 12.52). Nearly half of all respondents were women (49.9%), and 52% identified as white. 

### 3.2. Provider’s Receptivity to MCED Test Use

Table 2 overviews the exploratory factor analysis related to the PCPs’ receptivity towards the use of an MCED test. All 11 items had means above neutral (3.0), with there being strong agreement to certain challenges regarding MCEDs (e.g., cost of medical follow-up, insurance coverage). One item, Q3, was removed due to a weak loading (0.25) and low communality (0.23). 

The two extracted components from the experimental questionnaire, receptivity and challenges, were significantly correlated (r = 0.124, *p* = 0.024). Interestingly, this relationship is positive, meaning agreement with statements regarding the challenges of using MCEDs (e.g., cost of testing, explaining false positives) are related to agreement with statements such as believing most providers would order an MCED test. 

### 3.3. Provider Perceived Competencies in Using MCED Testing

Table 3 overviews the PCPs’ perceived competencies in using MCED testing. All five-item means were above neutral (3.5–3.9). As hypothesized, perceived competence in ordering MCED tests and managing results was strongly correlated with PCP receptivity to using MCED tests (r = 0.481, *p* < 0.001).

### 3.4. Variables Predicting Provider Receptivity to Use of MCEDs

Table 4 shows the scale scores and internal reliability for our primary outcome, PCP receptivity, as well as our secondary outcomes: awareness of challenges and perceived competence. All measures showed acceptable internal reliability (all Cronbach’s Alpha > 0.70).

Table 5 overviews the results of regression modeling to examine the variables associated with the PCP receptivity to use of MCEDs. Both perceived competence and provider experience had significant positive associations with receptivity to using MCEDs (*p*s < 0.001). 

### 3.5. Provider Characteristics

Provider characteristics were analyzed to determine if there were differences in providers’ receptivity to MCED use, awareness of challenges to MCED use, and perceived competence in using MCEDs. There was a statistically significant difference in the provider receptivity score between the three provider experience levels (F (2,300) = 3.52, *p* = 0.031). The mean receptivity score was higher for staff physicians with over 18 years in practice than resident and fellows (*p* = 0.022). Staff physicians were also categorized by their specialty, with respondents identifying as either internal or family medicine. Family medicine doctors were found to have higher perceived competence in using MCED tests than internal medicine doctors (t (114) = 2.10, *p* = 0.038). No differences were found in their awareness of challenges to MCED testing (*p* = 0.429) or receptivity to MCED testing (*p* = 0.060).

## 4. Discussion

The current study assessed the receptivity to MCED testing among a diverse group of providers including staff physicians, residents and fellows, and APPs in primary care. Although there has been a call for incorporating MCED testing into routine cancer screening, there is currently only one clinical assay available—Grail Galleri [10]—and little is known about how this new testing will impact primary care [21]. We found that PCPs are generally receptive to the idea of incorporating MCED testing into their routine practice for cancer screening. This is in contrast to previous research that found that PCPs had concerns about potential problems associated with genetic testing as a screening tool [22]. In particular, they reported insufficient confidence in their ability to order genetic testing and uncertainty around the clinical benefits of this testing as a screening method in low risk patients [12]. In contrast with other genetic testing, MCEDs appear to have a more clearly defined place in the practice of primary care [23]. MCED testing may represent a role for genetic testing for which PCPs can better understand the management of the results and their ability to explain them to their patients.

In fact, we found that PCPs had high levels of confidence in their ability to manage patients through every stage of MCED testing. This process included both educating them about MCED testing and then engaging them in shared decision making about whether or not they would want to have the test performed. This confidence is surprising since evidence shows that, although shared decision-making (SDM) is considered an important part of patient centered care, SDM is not common in clinical practice [24,25]. Reasons suggested for this include insufficient training in SDM and insufficient time during a patient visit. The respondents also expressed confidence in their ability to explain “false positive” and “false negative” test results and to guide patients with an abnormal test result through follow-up diagnostic testing. There are currently no clear answers to what the test properties will be for the various MCED tests when used for screening a population of asymptomatic adults. In an observational study of symptomatic adults who had an MCED test and a diagnostic outcome (SYMPLIFY), there were 368 (6.7%) cancer diagnoses in the final cohort of 5461 patients. The MCED test detected a cancer signal in 323 patients of whom 244 were diagnosed with cancer for a positive predictive value of 75.5% and sensitivity of 66.3% [12]. In an asymptomatic population using a different test (DETECT-A), the sensitivity of the test was 15.6% and the positive predictive value was 28.3%. A further analysis of the subjects with false positive results in the study provided some reassurance. These 98 women were followed for a total 4 years and only 2 developed cancer [26]. PCPs deal with uncertainty every day in their practice and those who completed this survey may have had some familiarity with the evolving knowledge of the test potential and limitations of MCEDs.

Not surprisingly, PCPs endorsed many of the items in the survey that related to potential challenges to the introduction of MCED testing into their practice. Several of these related to the amount of time that a discussion of MCED testing and the handling of the results would likely impose on an already busy patient schedule. Others related to concerns about the patient. These included whether patients would complete the additional testing associated with a positive test result and whether insurance would cover these recommended tests and procedures. Previous research has shown that patients often fail to complete recommended follow-up after a positive finding on conventional screening [27] and that these delays may result in a new cancer diagnosis [28]. The cost of the currently available MCED test alone will be outside the reach of many patients [13]. Determining the overall costs to the healthcare system and who should pay will need to involve health policy experts, insurers, and other constituents [29]. Primary care providers have very real concerns about the limited amount of time they have during a patient visit to complete all the tasks expected of them and they try to limit unnecessary costs and anxiety associated with unproven testing. What was surprising was that a high score on the awareness of challenges factor was associated with a higher level of receptivity to MCED testing. It may be that providers who have carefully thought through the process of MCED testing have decided that testing has value to their patients, even after considering the potential “costs”, financial and emotional.

Clinical experience also was related to physicians’ receptivity to incorporating MCED testing into their practice of cancer screening. Physicians with the greatest number of years in clinical practice reported the highest levels of receptivity to MCED testing while trainees were the least receptive to MCED testing. It is not clear what caused this difference. It may be that more experienced physicians are more comfortable negotiating the uncertainties of this new test modality with their patients, but this was not tested.

Interestingly, family medicine physicians reported a higher perceived competence with respect to MCED testing than those trained in internal medicine. As MCED testing is a new technology, it is unlikely that either group has had significant clinical experience in ordering MCED testing or managing the results. However, physicians from both disciplines should have similar exposure to the science behind MCED testing. That said, the reason for the higher perceived competence with respect to MCED testing by family physicians is unclear at this time. Regardless, it is anticipated that MCED testing will be performed as part of a comprehensive cancer screening plan which may make it more relevant to family physicians in primary care. Therefore, this higher perceived competence is encouraging but does require more investigation into the drivers. 

There are several limitations to this study. First, although the PCPs in this study are a large, diverse group, they all practice in a single regional health system. Secondly, the response rate of 37.4% is below the average response rate of physicians to web-based surveys (46%) [30]. We did not provide incentives, but it may be that the responders were more familiar with MCEDs. Therefore, these results are not generalizable to other healthcare organizations or provider groups. In addition, we asked participants to respond to a hypothetical scenario in which MCED testing had been approved for use in clinical care. As a result, we could not provide specific details about test performance (e.g., sensitivity, specificity, and positive predictive value), the integration of MCED testing with standard of care cancer screening, and insurance coverage. Thus, PCP’s individual assumptions related to the test characteristics and performance may have influenced their responses to survey items. Also, the timing of our surveys may have had an effect on the results. We surveyed trainees in a period of time that tended to follow the distribution of surveys to the staff physicians, many of whom were faculty for the residents and fellows. It is possible that trainees’ responses were influenced by discussions with other practitioners.

In summary, we found that PCPs in the study were generally receptive to the idea of incorporating MCED testing into their practice of screening for cancer. While they acknowledged the potential challenges to using MCED testing and the additional time that they would need to spend on ordering MCED testing and managing the results, the respondents signaled that they were receptive to MCED testing for cancer screening. Introducing MCED testing into routine screening for cancer will likely mean that the visits with the PCP will take longer or that other trained staff will need to be involved in the patient education process. Future research should focus on the process of shared decision making in the primary care provider’s office before MCED testing is ordered. It may be useful for providers to go through different scenarios with the patient including what might happen with a positive test result but a negative subsequent work up for cancer [21]. These false positive test results are likely to cause anxiety in both the patient and the provider. Previous experience has shown that new technology in cancer screening and treatment increases disparities in outcomes as a result of unequal application [31,32,33]. Primary care providers as advocates for their patients will be an important part of the plan for implementing MCED testing into routine practice. Keeping PCPs updated on the emerging knowledge in this area is likely to affect their confidence in ordering and managing MCED testing and results.

## Figures and Tables

**Table 1:** Demographics of survey respondents.

	Staff Physicians (*n* = 126)	Residents and Fellows (*n* = 201)	APPs (*n* = 24)
Age, Mean (sd)	50.8 (11.3)	30.8 (3.8)	44.6 (13.2)
Gender (%)			
Female	55 (43.7%)	102 (50.7%)	18 (75.0%)
Male	58 (46.0%)	84 (41.8%)	6 (25.0%)
Other/Unknown	13 (10.3%)	15 (7.5%)	0
Race			
White	85 (67.5%)	81 (40.3%)	17 (70.8%)
African American	3 (2.4%)	14 (7.0%)	2 (8.3%)
Latino/Hispanic	8 (6.3%)	28 (13.9%)	0
Asian	15 (11.9%)	61 (30.3%)	5 (20.8%)
Other/Unknown	15 (11.9%)	17 (8.5%)	0
Provider Specialty			
Family Medicine	73 (62.4%)	-	-
Internal Medicine	44 (37.6%)	-	-
Physician Experience			
Trainee	-	201	-
Under 18 years	59	-	-
Over 18 years	55	-	-

**Table 2:** ‘Provider Receptivity to MCED Test Use’ questionnaire items, means, and loadings.

		Factor Analysis ^1^	
Question	μ	Receptivity	Awareness of Challenges
Q9. I think most providers would order MCED testing for cancer screening.	3.53	0.844	
Q10. I believe MCED testing would improve cancer screening.	3.77	0.769	
Q5. I think most patients would be willing to do an MCED blood test for cancer screening.	3.59	0.630	
Q4. I believe that most patients would complete diagnostic follow-up for an abnormal MCED blood test result.	3.65	0.481	
Q7. I am worried about managing false positive MCED test results.	3.82		0.927
Q8. I am concerned about managing false negative MCED results.	3.73		0.793
Q1. I believe it would take a lot of time to explain MCED testing to patients.	3.59		0.525
Q11. I am concerned about the cost of diagnostic follow-up for patients who have an abnormal MCED test result.	4.04		0.514
Q6. I am concerned about whether patients would have insurance coverage for MCED testing.	4.24		0.400
Q2. I think I would have to know more about MCED testing before ordering it for my patients.	4.31		0.391
Q3. I am concerned that patients who do MCED testing would not complete standard of care cancer screening, such as mammography and colonoscopy. ^2^	3.43		

^1^ The eigenvalues and % of variance explained for the “Receptivity” factor were 2.26 and 22.60%, and for the “Challenges” factor they were 3.03 and 30.26%. All items were on scored a 5-point Likert scale (1—Strongly Disagree, 5—Strongly Agree). ^2^ The coefficient loadings show two clear factors with all items loading in either Receptivity or Awareness of Challenges with the exception of Q3.

**Table 3:** ‘Provider Perceived Competence in Using MCED Tests’ questionnaire.

Question ^1^	μ
C1. I would be confident in my ability to educate patients about MCED testing for cancer screening.	3.56
C2. I would be confident in my capacity to explain false positive MCED blood test results to patients (e.g., a positive MCED blood test result that is not confirmed upon further diagnostic testing).	3.67
C3. I would be confident in my ability to engage patients in shared decision making about MCED blood testing.	3.90
C4. I would be confident in my capacity to explain false negative MCED blood test results to patients (e.g., a negative MCED blood test result followed by a cancer diagnosis upon further diagnostic testing).	3.61
C5. I would be confident in my ability to guide patients with an abnormal MCED blood test result through follow-up diagnostic testing	3.63

^1^ All items were scored on a 5-point Likert scale (1—Strongly Disagree, 5—Strongly Agree).

**Table 4:** Scale score descriptives and reliability.

	Receptivity	Awareness of Challenges	Perceived Competence
Mean Score ^1^	3.62	3.95	3.67
Std. Deviation	0.66	0.64	0.85
Cronbach’s Alpha	0.75	0.77	0.91

^1^ Scale scores were averaged to fit the 5-point Likert scale response.

**Table 5:** Multiple regression predicting provider receptivity to the use of MCEDs.

Predictor ^1^	Unstandardized Coeff.		Standardized Coeff.	t	*p*
	B	Std. Error	Beta		
Perceived Competence	0.393	0.038	0.513	10.437	<0.001
Provider Experience	0.145	0.041	0.173	3.523	<0.001

^1^ Constant = 1.930, F (2,298) = 58.68, *p* < 0.001, R^2^ = 0.283.

## Data Availability

Data available upon request.
